# Exencephaly–Anencephaly Sequence Associated with Maxillary Brachygnathia, Spinal Defects, and Palatoschisis in a Male Domestic Cat

**DOI:** 10.3390/ani13243882

**Published:** 2023-12-17

**Authors:** Simona Marc, Jelena Savici, Bogdan Sicoe, Oana Maria Boldura, Cristina Paul, Gabriel Otavă

**Affiliations:** 1 Faculty of Veterinary Medicine, University of Life Sciences “King Mihai I” from Timisoara, Calea Aradului 119, 300645 Timisoara, Romania; simona.marc@usvt.ro (S.M.); jelenasavici@usvt.ro (J.S.); bogdan.sicoe@usvt.ro (B.S.); oanaboldura@usvt.ro (O.M.B.); gabrielotava@usvt.ro (G.O.); 2 Department of Applied Chemistry and Engineering of Organic and Natural Compounds, Faculty of Industrial Chemistry and Environmental Engineering, Politehnica University Timisoara, Carol Telbisz 6, 300001 Timisoara, Romania

**Keywords:** exencephaly, anencephaly, congenital malformations, neural tube defects, feline

## Abstract

**Simple Summary:**

A stillborn male kitten from an accidental inbreeding was examined through different paraclinical methods, such as radiographic, CT, and cytohistological examination, due to the existence of severe neural tube defects associated with other congenital defects. The malformations observed included exencephaly–anencephaly, closed cranial spina bifida at the level of cervical vertebrae, maxillary brachygnathia, kyphoscoliosis, palatoschisis, and partial intestinal atresia. These severe malformations have a multifactorial cause in which both genetic and environmental factors can intervene. Inbreeding increases the possibilities of genetic defects to be expressed in the phenotype.

**Abstract:**

Anencephaly, a severe neural tube defect characterized by the absence of major parts of the brain and skull, is a rare congenital disorder that has been observed in various species, including cats. Considering the uncommon appearance of anencephaly, this paper aims to present anencephaly in a stillborn male kitten from an accidental inbreeding using various paraclinical methods. Histological examination of tissue samples from the cranial region, where parts of the skull were absent, revealed the presence of atypical nerve tissue with neurons and glial cells organized in clusters, surrounded by an extracellular matrix and with an abundance of blood vessels, which are large, dilated, and filled with blood, not characteristic of nerve tissue structure. In CT scans, the caudal part of the frontal bone, the fronto-temporal limits, and the parietal bone were observed to be missing. CT also revealed that the dorsal tubercle of the atlas, the dorsal neural arch, and the spinal process of the C2–C7 bones were missing. In conclusion, the kitten was affected by multiple congenital malformations, a combination of exencephaly–anencephaly, maxillary brachygnathism, closed cranial spina bifida at the level of cervical vertebrae, kyphoscoliosis, palatoschisis, and partial intestinal atresia. The importance of employing imaging techniques cannot be overstated when it comes to the accurate diagnosis of neural tube defects.

## 1. Introduction

Neural tube defects (NTDs) are a group of severe congenital malformations of the nervous system caused by incorrect or incomplete closure of the neural tube during embryonic development [1]. There are two main ways in which the neural tube is formed from the neural plate during embryonic development: primary neurulation for the brain and most of the spinal cord, and secondary neurulation for the sacrum and tail bud [2,3]. 

In *primary neurulation*, the cells surrounding the neural plate communicate with the cells themselves, determine to proliferate, invaginate inward, and finally detach from the outer surface. This process of invagination and separation gives rise to a hollow tube, the neural tube, which extends along the longitudinal axis of the embryo [2]. The segments formed through primary and secondary neurulation later join together at the future upper sacral level to form the entire neural tube [2,3,4].

During this complex embryonic development, if the cephalic folds of the neural tube do not fuse properly, it will cause the absence of ectodermal tissue which should form the skeletal and muscular structures that cover the underlying neural structures. Due to this maldevelopment, the skull is partially missing and the neural structures remain open, defined as exencephaly. Although the brainstem, cerebellum, and spinal cord are present, only part of the diencephalon may be preserved [5,6,7]. Due to the exposure of the brain tissue to the amniotic fluid, which causes degeneration and neural deficits, clinical aspects of anencephaly can be seen. This condition is fatal within hours after birth [5].

Anencephaly is reported rarely on OMIA (“Online Mendelian Inheritance in Animals”, a database intended to list all hereditary diseases with simple determinism) in calves (OMIA:000044-9913) [8,9,10], sheep (OMIA:000044-9940) [11], greater Indian rhinoceros (*Rhinoceros unicornis*) (OMIA:000044-9809), and Indo-pacific bottlenose dolphins (*Tursiops aduncus*) (OMIA000044-79784) [12], but also in bears [13] and dogs [14,15,16,17,18]. In dogs, in almost all cases, puppies affected by anencephaly also presented with other malformations such as brachygnathia [14], atresia ani, hydrocephalus [16], gastroschisis, amelia of the right anterior limb [17], cleft palate [15], or cleft palate and cleft tongue [19]. Some rare cases with severe NTDs were reported in captive wild animals, such as a horned rhinoceros (*Rhinoceros unicornis*) [20], and a brown bear (*Ursus arctos arctos*) [13], where the etiology is more complicated to identify because these animals are kept under strict health control, so the influence of environmental factors is minimal, although possible nutritional deficiencies, especially in folic acid, are proposed.

In the veterinary literature, the main forms of NTDs reported other than anencephaly are encephalocele, craniorachischisis, dermoid sinus, and spina bifida [1]. These forms of NTDs are classified as *cranial dysraphism* (e.g., anencephaly, encephalocele, craniorachischisis) and as *spinal dysraphism* (e.g., spina bifida cystica, spina bifida occulta). Congenital malformations of the nervous system can affect only the nervous system or also involve surrounding tissues such as bone, muscle, and connective tissue [21]. 

Spinal dysraphisms are a diverse category of congenital disorders involving the spine and spinal cord that result from abnormal formation of midline mesenchymal, bony, and neural structures. Additionally, neural tube defects can be subdivided into ventral or dorsal midline defects, depending on the location of the missed closure. They can be *open spinal dysraphism* (e.g., open spina bifida or myeloschisis, myelomeningocele, myelocele, hemimyelocele and hemimyelomeningocele), when the lesion is exposed to the environment without skin covering and *closed spinal dysraphism* (e.g., closed spina bifida, lipomyelocele, lipomyelomeningocele, etc.), when the lesion is covered by the surrounding skin, or without subcutaneous mass (e.g., dermoid sinus, etc.), orifices, dyschromia, angiomas, or tufts of hair. Most of the open and closed spinal dysraphisms are located in lumbar or thoraco-lumbar regions, followed by lumbosacral regions and finally those from cervical and thoracic regions [5,22]. 

Anomalies of the spinal cord and/or other systems, particularly those originating in the early embryonic developmental period, may be associated with congenital anomalies of the vertebral column [23]. This link between the spine and spinal cord abnormalities can be explained by the fact that their development occurs side by side. During embryonic development, the vertebral column is formed from somites, which are temporary paired segments from the paraxial mesoderm surrounding the neural tube in the early embryo. Following this process, the dorsal part of the somite forms the dermomyotome, which will differentiate into muscle and dermis, while the ventral part forms the sclerotome. The migration of the sclerotomes around the neural tube and the notochords leads to the formation of vertebral bodies, arches, transverse, and spinous processes [24]. This process is governed by a genetic mechanism that includes many signaling pathways (Notch, Wnt/beta-catenin, and Fibroblast growth factors), and disruption of these mechanisms have been reported as the cause of vertebral segmentation defects in humans [25,26]. 

Palatoschisis or cleft palate (CP) (OMIA 000197-9685), another malformation identified in our case, is a developmental defect of the palate resulting from a failure of the medial fusion of the palatine processes and manifesting as a separation of the hard or/and soft palate. It has been reported in cattle [27,28], dogs, especially brachycephalic breeds (4), horses [29], cats [30], wild animals (lion, jaguar, tiger) [31,32], and even spectacled flying foxes [33]. Among the human population, palatoschisis with or without cheiloschisis is the most commonly occurring craniofacial birth defect, with a variety of model organisms, such as mice and dogs, utilized to understand the development of orofacial clefts.

Compared to human medicine, the description of such congenital defects is still poor in veterinary medicine. To increase knowledge about NTDs in the feline species, the aim of this article is to describe the clinical, radiographic, CT, histopathological, and dissection findings in one kitten affected by exencephaly–anencephaly, closed cervical dysraphism, kyphoscoliosis, maxillary brachygnathism, palatoschisis, and partial intestinal atresia.

## 2. Materials and Methods

### 2.1. Animal

The stillborn male kitten described here along with two other kittens were the offsprings of a 3-year-old domestic cat. The kittens were the result of an accidental inbreeding, between daughter and father. The cat had no previous parturitions. The kittens were delivered through a hysterotomy conducted in the Small Animal Reproduction Clinic from Faculty of Veterinary Medicine, Timisoara on 7 May 2022. The anesthesia protocol was carried out with Xylazine (Bioveta, Ivanovice na Hané，Czech Republic) at 1.2 mg/kg body weight IM, Ketamine (Ketamita 10%, Richter Pharma, Wels, Austria) at 10 mg/kg body weight IM, and Propofol (Propofol-Lipuro, Braun, Melsungen, Germany) at 1–2 mg/kg body weight administrated IV. One of the kittens displayed distinctive manifestations of neural tube defects described in Section 3.1, while the other two had no congenital malformations and were alive.

### 2.2. Cytohistological Investigation

Tissue samples were prepared for paraffin (paraffin for histology, Merck, Germany) embedding. The hematoxylin–eosin method was used for the staining of the slides, which were examined on an Olympus CX41 microscope (Olympus, Hamburg, Germany) using QuickPHOTO Micro 2.2 analysis software (Promicra, Prague, Czech Republic).

### 2.3. Computed Tomography Investigation

The acquisition of CT (Computed Tomography) scan images was performed with the body positioned in a lateral recumbency. CT scans were performed with a Siemens Somatom Definition AS 64 scanner, using conventional settings (90 kV, 100 mAs), and a slice thickness of 0.6 mm.

The computer tomograph images that were under the form of VRT Imagines (Volume Rending Technology) and MPR Imagines (Multiplanar reconstruction images) were analyzed using the CT’s own DICOM viewer, as well as a MicroDICOM viewer.

### 2.4. Radiography Investigation

Radiographic (Rx) images of the stillborn kitten were acquired with the Siemens Multix Swing digital CR system using the following settings: 52 kV and 12.5 mAs. The kitten was placed on the radiographic table in right lateral recumbency.

### 2.5. Dissection Investigation

After Rx, CT and histological sample, the specimen was prepared to investigate specific internal features. It was fixed in 9% non-buffered formalin and after a period of time internal structures (vital organs) were examined. 

## 3. Results

### 3.1. Clinical Presentation

**Figure 1:**
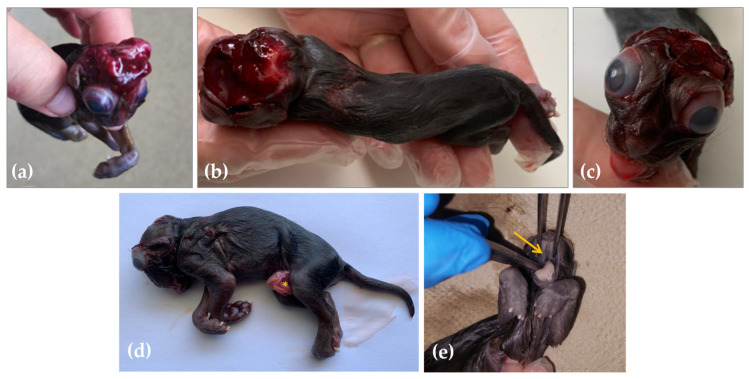
Head, dorsal view, stillborn kitten fetus. The brain tissue was covered by a reddish membrane (**a**). The whole body of the kitten, dorsal view, scoliosis is visible (**b**). Head, rostral view, shallow orbits with protrusion of the globes, and maxillary brachygnathia (**c**). Lateral view with the umbilical cord with a segment from the colon visible (marked with *) (**d**). Palatoschisis, severe form—marked with an arrow (**e**).

The skull of the stillborn male kitten showed a flattened morphology with incomplete closure of the dorsal part (Figure 1). Consequently, an irregular reddish membrane covered the underdeveloped cranial cavity. Furthermore, the skull base appeared abnormally thickened and flattened compared to typical specimens. The shallow orbits gave rise to ocular protrusion. Caudally to the base of the skull, skin was present. The maxilla exhibited brachygnathia, a well-known condition characterized by abnormal jaw alignment. Furthermore, the vertebral column showed features of kyphoscoliosis combined with lordosis. 

**Figure 2:**
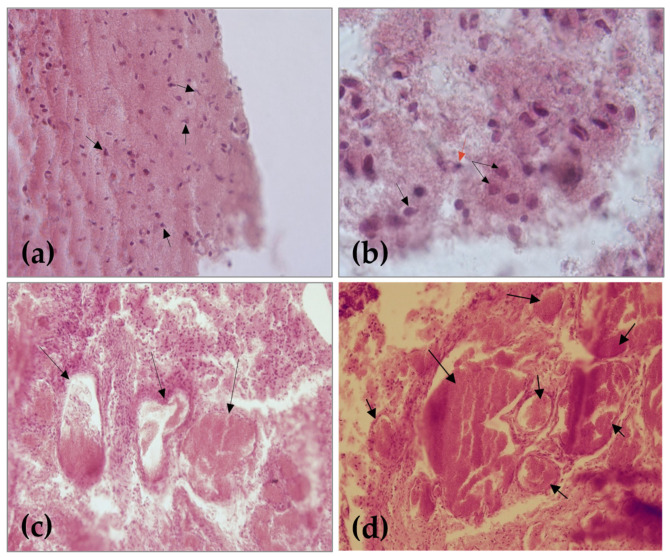
Histological sections: (**a**) **←** neurons, H.E. staining, 40× ob; (**b**) **←** neurons, **←** glial cells, H.E. staining, 100× ob; (**c**) **←** blood vessels, H.E. staining, 20× ob; (**d**) **←** large blood vessel with various diameters, 20× ob.

Histological examination of tissue samples from the cranial region, where the skull was absent, revealed the presence of nerve tissue. Physiologically, nerve tissue consists of extracellular matrix and cells: neurons and glial cells. The perikaryon of neurons together with the glial cells form the grey matter and their extensions the white matter. The two substances have a characteristic arrangement in each segment of the brain. However, microscopic examination of samples revealed loss of the normal structure and the presence of atypical nervous tissue. The irregular mass of the neuroglial tissue consists of the neurons or neuroblasts and glial cells, even though there were cases in which these cells were absent. Nerve cells are organized in clusters, surrounded by an extracellular matrix, without evidence of specific differentiation of the grey and white matter (Figure 2a,b). 

The tissue mass with poorly outlined architecture also revealed an abundance of blood vessels, which are large, dilated, and filled with blood. An angiomatous mass represented by the enlarged blood vessels of varying diameters, surrounded by connective tissue and nervous tissue islands, is a typical aspect found in anencephaly (Figure 2c,d).

**Figure 3:**
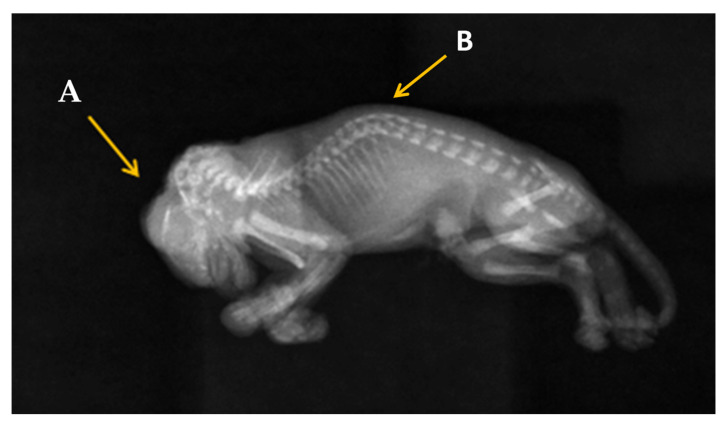
Radiographic image of the kitten: (A) Partial absence of skull bones, (B) kyphoscoliosis. There is no abdominal serosal detail due to lack of abdominal fat. The gastrointestinal gas is not visible to provide a contrast.

In Figure 3, which is a complete radiographic image on the entire kitten in right lateral recumbency, we can observe that the main caudal parts of the skull are missing, such as caudal part of the frontal bone, the fronto-temporal limits, and the parietal bone. The cervical dysraphism cannot be observed on this radiographic image. An abnormal curve of the spine is observed in the thoracal region, which is referred to as kyphoscoliosis. 

**Figure 4:**
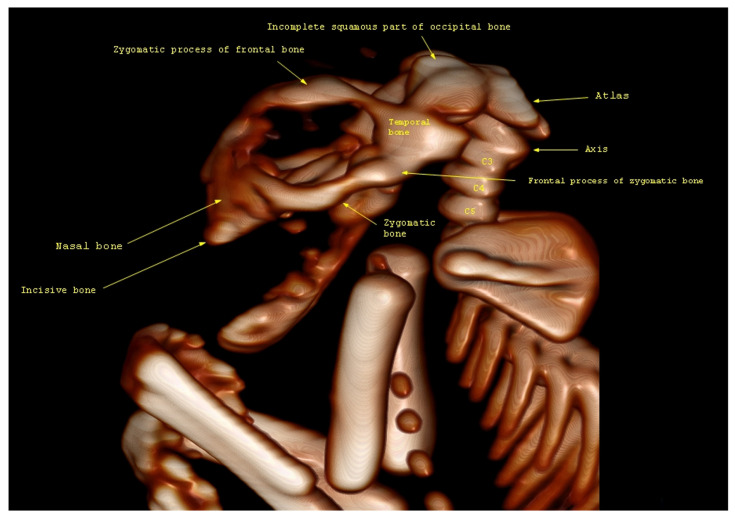
CT scan with VRT images of the kitten’s skull.

CT images were examined to identify any abnormalities in the kitten’s brain and spinal structures. The skull’s dimensions, the presence of brain malformations (such as partial or total absence of the brain), and the presence of defects in the cervical spine were analyzed. In addition, any associated abnormalities such as brachygnathia or other congenital malformations were assessed. From the CT analyses, it is seen that the caudal part of the frontal bone is missing, as well as the fronto-temporal limits and parietal bones. The other bones of the skull, such as the zygomatic process of the frontal bone, the frontal process of the zygomatic bone, the nasal and incisive bone, some parts of the occipital bone, and the occipital condyles are present (Figure 4).

**Figure 5:**
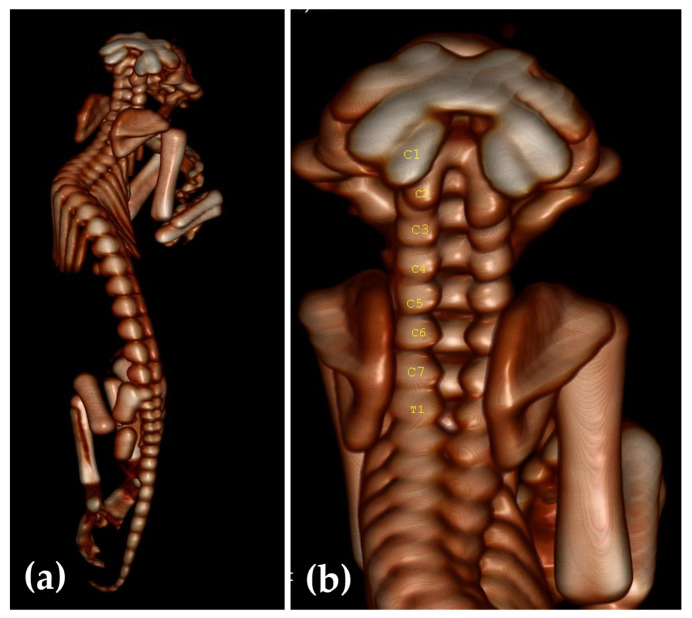
CT with VRT images of the kitten: (**a**) Complete spine image, (**b**) cervical vertebra with no dorsal arches/lamina.

Complete CT examination of the spine (Figure 5a) revealed kyphoscoliosis. In Figure 5b it can be observed that the dorsal neural arch and the dorsal tubercle of the atlas (C1) are missing, but the transversal processes are present. The dorsal neural arch and the spinal process of C2–C7 are also missing. The fact that the dorsal arches of cervical vertebrae were missing led to a retroflexion of the spine. Also, the first thoracic vertebra had its dorsal arch incompletely closed. 

**Figure 6:**
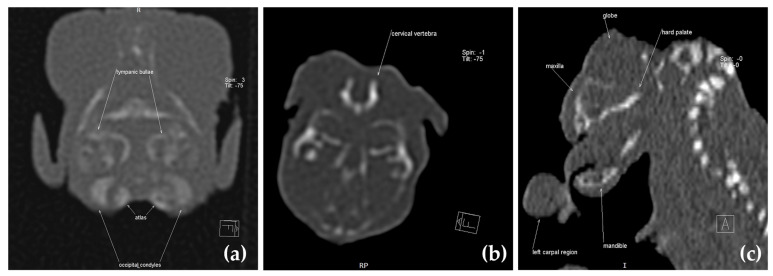
CT scan with MPR images of the skull. (**a**) Tympanic bulla, first cervical vertebral, atlas and occipital condyles; (**b**) first cervical vertebral and atlas; (**c**) the eye, maxilla (more exactly, nasal bone), palate bone, and mandibula.

In Figure 6, we can see on the multiplanar reconstruction (MPR) images the main anatomical segments identified as follows: (a) tympanic bulla, first cervical vertebral, atlas, and occipital condyles; (b) first cervical vertebral and atlas; (c) the eye, maxilla (more exactly, nasal bone), palate bone, mandibula, and parts from left carpal region.

The macroscopic findings of the absence of brain parts, hypoplastic calvarium, thickened skull base, and identifiable cranial nerves and fragments of cerebellar folia observed in the feline fetus reported here closely resemble the characteristic features of anencephaly. The fact that the dorsal arches of the cervical vertebrae are not closed can lead to spinal flexion, as can be seen also in this case. In human medicine, exaggerated spinal retroflexion observed in most CNS malformations is due to the absence of neural arches [34]. The kitten’s condition was confirmed by a CT scan, which indicated exencephaly–anencephaly, closed cranial dysraphism affecting the cervical vertebrae C1–C7 and T1, and thoracal kyphoscoliosis. 

At dissection examination, a portion of the duodenum was protruding through the umbilical ring as well as the transverse colon, the kidneys were normal, the liver increased in volume and occupied the abdominal cavity, and the spleen, stomach, and kidneys were present. In the thoracic cavity, the lungs and heart did not show any visible changes.

## 4. Discussion

The clinical examination of the kitten showed partial absence of skull bones with exposure of nervous tissue as a reddish, indistinguishable tissue, signs specific in exencephaly–anencephaly. Other signs identified at the head were the ocular protrusion with shallow orbits, the maxilla was shorter than normal, signs of brachygnathia, and, after a careful examination of the oral cavity, a complete cleft palate. The ocular protrusion, so called “frog eye” sign, is a consequence of the absence of the fetal calvarium. Normal vision in such cases is affected due to retinal ganglion cells reduction and optic nerves abiotrophy as a sequel of secondary retrograde degeneration [14,19]. Histological results conducted on brain tissue described atypical nerve tissue with an abundance of large, dilated, and filled blood vessels, named area cerebrovasculosa, seen also in the literature in both human and animal cases with anencephaly in which the lack of encephalon was not total [4,7,14,35]. The abnormal microscopic aspect of nerve tissue probably resulted from the degeneration of previously formed neural tissue due to exposure to amniotic fluid.

In felines, the main and relatively uncommon NTDs reported are meningocele, meningoencephalocele [36,37], myeloschisis [38], and spina bifida. Burmese cats are predisposed to craniofacial anomalies due to their high linkage disequilibrium and inbreeding. The craniofacial defect is characterized by meningoencephalocele, agenesis of all derivates of the medial nasal prominence, and lateral duplication of most derivatives of the maxillary process. This defect has a simple autosomal recessive mode of inheritance and seems that a highly popular sire in the USA, used in reproduction due to his extreme brachycephalic phenotype, was responsible for the defect [39,40].

Similar dog cases are reported in the literature; thus, a case of anencephaly in the Pomeranian breed presented the lack of cerebral hemispheres in cavum cranii, incomplete skull formation, and exophthalmos, associated with tongue split and palatoschisis [19]. Another case with anencephaly associated with cranioschisis was reported in Pincher breed [18]. In all cases, the puppies were stillborn [14,19], died soon after parturition [18] or were sacrificed soon after birth due to the severe malformations [15], being unable to suckle and to coordinate. Cases of anencephaly have also been reported in wildlife, captive or free-ranging (bears, dolphins). Data on congenital malformations (affecting the CNS) in wild animals are difficult to observe and remain unrecorded because it is difficult to find stillborn animals in nature [13].

Regarding the gender predisposition to NTDs, in veterinary medicine it is not known, probably due to low number of reported cases (most of the articles cited in this paper were females) [13,18,19], but a female predisposition has been reported in human medicine, possibly because of a sex-related genetic effect or an epigenetic effect [4] but not due to the different rate of embryonic growth and development between females and males [12].

The etiology of congenital malformations is quite difficult to realize, because there are a lot of factors, both genetic and environmental, that can produce phenocopies, or the cause can be a consequence of the interaction of genetic and exogenous factors. Etiology for NTDs is poorly reported in the veterinary medical literature, but from human medicine it is known to be multifactorial because genetic factors or/and environmental factors can cause CNS malformations, therefore a multifactorial pattern is supposed to be incriminated [5,21,41].

The embryonic development of the neural tube is a multistep process that is controlled by many genes, with more than 200 genes identified in mice that are involved in the formation of the neural tube [1]. Some of these genes are evolutionarily highly conserved, and their role in neurulation has been shown in multiple vertebrate animal models [4]. Gene mutations have been identified as risk factors in the mouse model with NTDs, from which genes related to mitochondrial folate metabolism, such as *MTHFD1L, AMT, SLC23A32*, or the *FOLR1* genes, are often incriminated [42,43,44,45,46,47,48,49,50]. Mutations in genes that control epigenetic activity, such as DNMT3B or abnormal epigenetic modification in *LINE-1*, *JARID2*, *FBXL10*, and *SIRT1* genes, have been reported to have clinically seen consequences as NTDs [51,52,53]. Based on a recent study in humans, genetic abnormalities explain only 10% of the acrania–exencephaly–anencephaly sequence, but with the help of genome-wide sequence information, the genetic background of these NTDs can be better understand [54]. 

Exogenous factors can be implicated in NTDs, such as: chemical toxins (e.g., organophosphorus pesticides, amide, benzimidazole, methyl carbamate) [55,56], metals (e.g., mercury, arsenic) [57,58,59], infectious agents, toxic plants, hypervitaminosis A [60], and even hyperthermia or ethanol [61]. Antiepileptic drugs (valproate, carbamazepine, phenobarbital) are associated with risk for NTDs, because their actions act as folate antagonists [35]. An important risk factor for NTDs is the deficiency in folic acid (vitamin B9), and laboratory animals with gene mutations in enzymes encoding mitochondrial folate metabolism express NTDs [45,46,48,49]. Folic acid in an essential nutrient for mammalian cell growth, being involved in the synthesis of essential components for fetal development, such as purine and pyrimidine, and in the conversion of homocysteine to methionine [62]. Because there were human cases in which maternal levels of folate were within the normal range and yet had affected products, it is clear that deficiency in folate can be a risk factor for NTDs, but there may also be a predisposing genotype [63]. In veterinary medicine, the additional administration of folic acid to pregnant animals is still poorly studied, although commercial products are available. Scientific data regarding the beneficial effect of oral folic acid supplementation are contradictory. Gonzales et al. [64] concluded that folic acid supplementation, from the onset of heat until the 40th day of gestation, does not decrease the risk of congenital malformations in dogs, while Domoslawska et al. [65] observed that folic acid supplementation decreased the incidence of palate and/or lip cleft in bitches.

There are different forms of NTDs for which differential diagnostics need to be conducted in anencephaly cases; for example, encephalocele, a significant congenital anomaly that affects the skull and brain which results from failure of the anterior neuropore to close [5,66]. In encephalocele, the meninges, with brain tissue, protrude towards the exterior through an opening in the skull as a sac, leading to the exposure of the brain and potential damage before and after birth [67]. Meningocele and meningohydroencephalocele are also skull base developmental defects that result in herniation of the dura and arachnoid (in meningocele) through a laminar defect in the spinal column [68], or the herniations of meninges, cerebrospinal fluid, brain parenchyma, and a small part of the ventricular system (in meningohydroencephalocele) [69]. While, in clinical terms, meningocele or meningohydroencephalocele is distinguished by a smaller skull defect with the sac-like protrusions being covered with skin, exencephaly–anencephaly is characterized by a massive skull defect and no skin over it.

Dermoid sinus is another NTD for which differential diagnosis needs to be conducted. The etiology of dermoid sinus is the failure of the neural tube to separate from the skin ectoderm during embryonic development, and clinically appears as a tubular sac extending from the dorsal midline deep inside other tissues [70,71]. This type of NTD identified in cats and dogs can be linked to vertebral malformations like spina bifida, hemivertebrae, block vertebra, or others [72]. The main difference between dermoid sinus and anencephaly is that histologically, in the dermoid sinus, epithelial cells and follicle structures with sebaceous and sweat glands are identified, and another difference is the location, dermoid sinus being located on the spinal cord [72]. 

After a closer examination of the oral cavity, complete palatoschisis was identified. Like NTDs described in this study, cleft palate is also considered a multifactorial pathology, involving genetic and environmental factors, or interaction between them. Its evolution is non-syndromic, or it can be syndromic with claw deformities, muscle weakness, and neurological signs. In cats, palatoschisis is not commonly described; only seven cases were reported prior to 2022 [30]. The palate malformation in animals presents a range of signs from a small pinhole cleft to a complete hard and palate deficit; in the kitten identified in this study, the cleft was complete. Genome-wide studies of dogs have identified a candidate gene for CL/P development as *ADAMTS20* (a disintegrin-like and metalloprotease with thrombospondin type-1 motifs) that is involved in cleaving extracellular matrix proteins and processing procollagen, being involved in palatogenesis, interdigital web regression, and in developing fore and hind limbs [73]. Due to the many molecular signaling pathways, such as *BMP*, *FGFs*, *SHH*, *WNT*, and others, involved in the growth and fusion of facial processes, and the possibility of environmental factors interacting with their function, it is very difficult to identify the genetic cause incriminated in the phenotype [74,75].

Some environmental factors known to induce cleft lip/palate (CL/P) are excessive doses of vitamin A (125,000 UI/kg BW at 17–22 days of gestation) or aspirin (400 mg/kg/day at 23–30 days of gestation) in bitches, various substances such as griseofulvin, anabasine, metronidazole, primidone, sulphonamides, and 6-diazo-5-oxo-L-norleucine (DON), viral infections, cytostatic drugs, stress, or hormonal factors [76,77].

During dissection examination, it was found that a part of the transverse colon was present at the level of the umbilical cord. The abdominal wall was closed, which indicates that it is not an omphalocele, where a defect persists in the abdominal wall and the abdominal content is covered by peritoneum. Likewise, we cannot speak of gastroschisis, because no visible abdominal mass outside the abdominal cavity was identified. To understand this anomaly, the physiological embryonic development of the digestive tube must be described. The gastrointestinal system is formed during the gastrulation process, mainly from the endoderm. Thus, the intestinal mucosa and digestive glands (liver and pancreas) are formed from the endoderm, the intestinal muscular layer, connective tissue, and blood vessels are formed from the lateral mesoderm, and the extremities of the gastrointestinal tube are formed from the ectoderm, through invagination, specifically the oral cavity and cloaca. Also from the ectoderm, more precisely from the neural crest, the enteric nervous system is formed [78,79]. During fetal development, physiological herniation of the midgut (which consists of mid duodenum, jejunum, ileum, cecum, and ascending and transverse colon) throughout the umbilical ring take place. It grows outside the peritoneal cavity, then rotates back and returns to the abdominal cavity, then the omphalomesenteric canal is obliterated, causing the connection between the midgut and the yolk sac to disappear [79].

A possible explanation of our findings is the one offered by Lejeune et al. [80], namely that the part of the intestine that normally herniates suffered ischemia at the point of exit and re-entry into the abdomen, which ended with the atresia of these segments.

Another congenital abnormality identified in this case was the lateral curvature of the spine in the thoracal region, visible clinically and accompanied by a sagittal deformity seen only on Rx image, so the abnormal vertebral development identified in the thoracal vertebrae was kyphoscoliosis. The thoracic segments of the spine are the most encountered region affected by kyphoscoliosis which results from abnormalities in vertebral chondrification centers [81,82]. Normally the chondrification centers appear within a mesenchymal template, which enclose the notochord and developing neural tube, and it is followed by ossification [24]. In our case, a leak of the dorsal neural arch and the spinal process of C2–C7 and T1 were observed, perhaps due to no migration of the sclerotomes around the dorsal part of neural tube and with no chondrification and ossification follow-up. This severe defect probably led to a retroflexion of the spine. Vertebral malformations can be seen in any breed, but some breeds of dogs, such as small brachycephalic breeds (e.g., Frech bulldogs, English bulldogs, pugs) [83], or some feline breeds, such as Manx cat, are more notable. In Manx cat, due to the absence of one or all coccygeal vertebrae, different types of neural malformation (e.g., spina bifida, syringomyelia, or even meningocele) can appear [84]. The pathophysiological mechanism in thoracic congenital malformations is also multifactorial, being associated with alterations in spinal biomechanics, that in the end can increase intervertebral disc extrusions [85].

The final diagnosis in this case was made based on Rx images, CT images, and histological and dissection examination. The etiology in this case has not been established, due to the fact that the anomalies of CNS have a multifactorial etiology, as explained above, making it difficult to identify. The mother had not received any medications during pregnancy, so drugs with a potential teratogenic effect are excluded. Due to the fact that the pregnancy was the result of incest (breeding between the daughter and the father), the high probability is that through incest, a mutant allele has a higher chance to express in the phenotype, especially those that are recessive, so it is possible for the male cat to be heterozygous for a serious of mutant genes.

In veterinary medicine, feline genomic data about such congenital malformations almost do not exist, although there are some clinical reports in the literature about the existence of these congenital pathologies. Therefore, despite the limitations, the present results provide a basis for further genomic investigation to identify the possible genes responsible for these changes; this will be our future research goal.

Based on the images from Rx, CT scan, and histopathological and dissection examination, the male kitten was affected by multiple congenital malformations, exencephaly–anencephaly, maxillary brachygnathism, closed spinal dysraphism at the level of the cervical segment, kyphoscoliosis, palatoschisis, partial intestinal atresia, and arthrogryposis on anterior legs. The literature review revealed no other previous cases with this combination of malformations in cats, so this case is the first reported.

## 5. Conclusions

Our findings presented in this article enhance the available data on animal exencephaly–anencephaly associated with many other congenital defects and underscore the importance of utilizing paraclinical investigations like Rx, CT, and histology to detect congenital anomalies in neonates and correctly diagnose NTDs. Due to the multiple genetic mutations that can cause these malformations, further in-depth studies are needed. The scientific literature only has limited information on the incidence rate of these malformations in small animals like domestic cats, which is why more data are needed in this field of animal congenital defects.

## Data Availability

Data are contained within the article.

## References

[1] Zarzycki A., Thomas Z.M., Mazrier H. (2021). Comparison of inherited neural tube defects in companion animals and livestock. Birth Defects Res..

[2] Hyttel P., Sinowatz F., Vejlsted M., Keith B. (2010). Essentials of Domestic Animal Embryology.

[3] Harrington M.J., Hong E., Brewster R. (2009). Comparative analysis of neurulation: First impressions do not count. Mol. Reprod. Dev..

[4] Avagliano L., Massa V., George T.M., Qureshy S., Bulfamante G.P., Finnell R.H. (2019). Overview on neural tube defects: From development to physical characteristics. Birth Defects Res..

[5] Salih M.A., Murshid W.R., Seidahmed M.Z. (2014). Classification, clinical features, and genetics of neural tube defects. Saudi Med. J..

[6] Golden J.A., Chernoff G.F. (1995). Multiple sites of anterior neural tube closure in humans: Evidence from anterior neural tube defects (anencephaly). Pediatrics.

[7] Monteagudo A. (2020). Exencephaly-anencephaly Sequence. Am. J. Obstet. Gynecol..

[8] Cho D.Y., Leipold H.W. (1978). Anencephaly in calves. Cornell Vet..

[9] Washburn K.E., Streeter R.N. (2004). Congenital defects of the ruminant nervous system. Vet. Clin. N. Am. Food Anim. Pract..

[10] Hiraga T., Abe M. (1986). Anencephaly and Other Congenital Defects in a Calf. Jpn. J. Vet. Sci..

[11] Dennis S.M., Leipold H.W. (1972). Anencephaly in sheep. Cornell Vet..

[12] Brook F. (1994). Ultrasound Diagnosis of Anencephaly in the Fetus of a Bottlenose Dolphin (*Tursiops aduncas*). J. Zoo Wildl. Med..

[13] Balseiro A., Polledo L., Tuñón J., García Marín J.F. (2022). Anencephaly and Severe Myelodysplasia in a Stillborn Brown Bear (*Ursus arctos arctos*). Animals.

[14] Huisinga M., Reinacher M., Nagel S., Herden C. (2010). Anencephaly in a German Shepherd Dog. Vet. Pathol..

[15] Luz M., de Mello Bobány D., Monteiro Silva M.E., Mouura Carvalho C.F. (2016). Anencephaly associated with cleft palate in a Bull Terrier litter: Case report. IOSR J. Agric. Vet. Sci..

[16] Thomas Z.M., Podadera J.M., Donahoe S.L., Foo T.S.Y., Weerakoon L., Mazrier H. (2020). Neural tube defects in four Shetland sheepdog puppies: Clinical characterisation and computed tomography investigation. Aust. Vet. J..

[17] Ortega-Pacheco A., Lezama-García M.A., Colín-Flores R., Jiménez-Coello M., Acevedo-Arcique C., Gutiérrez-Blanco E. (2020). Presence of congenital anomalies in three dog litters. Reprod. Domest. Anim..

[18] Nonato I.d.A., Teixeira M.R., de Miranda J.L., Bressan Braz H.M., Machado J.P. (2019). Cranioschisis and Anencephaly in a Dog—Challenging etiology. Acta Sci. Vet..

[19] Yavaş Ö., Yavaş S.E., BaşAr D., Avci Z., Sariçetin A., Yildiz E.R., Ersoy S., Özyiğit Ö. (2023). Anencephaly, Bifid Tongue, and Cleft Palate in a Pomeranian Dog: GFAP and NeuN Immunoreactivities. Ank. Üniv. Vet. Fakültesi Derg..

[20] Schaftenaar W., Fernandes T., Fritsch G., Frey R., Szentiks C.A., Wegner R.D., Hildebrandt T.B., Hermes R. (2011). Dystocia and Fetotomy Associated with Cerebral Aplasia in a Greater One-horned Rhinoceros (*Rhinoceros unicornis*). Reprod. Domest. Anim..

[21] Greene N.D.E., Copp A.J. (2014). Neural Tube Defects. Annu. Rev. Neurosci..

[22] Franco G.G., Siqueira E.G.M., Souza J.A.L., Prado L.O.C., Rahal S.C., Mamprim M.J., Minto B.W., Brandao C.V.S., Costa J.S. (2021). Closed spinal dysraphism in a 6-month-old mixed breed dog. Vet. Med..

[23] Westworth D.R., Sturges B.K. (2010). Congenital Spinal Malformations in Small Animals. Vet. Clin. N. Am. Small Anim. Pract..

[24] Chaturvedi A., Klionsky N.B., Nadarajah U., Chaturvedi A., Meyers S.P. (2018). Malformed vertebrae: A clinical and imaging review. Insights Imaging.

[25] Eckalbar W.L., Fisher R.E., Rawls A., Kusumi K. (2012). Scoliosis and segmentation defects of the vertebrae. WIREs Dev. Biol..

[26] Pourquié O. (2011). Vertebrate Segmentation: From Cyclic Gene Networks to Scoliosis. Cell.

[27] Reinartz S., Hellige M., Feige K., Wenning P., Distl O. (2015). Phenotypic classification of variability of non-syndromic congenital cleft lip and jaw in Vorderwald × Montbéliarde cattle. Acta Vet. Scand..

[28] Lupp B., Reinhardt M., Maus F., Hellige M., Feige K., Distl O. (2012). Right-sided cleft lip and jaw in a family of Vorderwald × Montbéliarde cattle. Vet. J..

[29] Shaw S.D., Norman T.E., Arnold C.E., Coleman M.C. (2015). Clinical characteristics of horses and foals diagnosed with cleft palate in a referral population: 28 cases (1988–2011). Can. Vet. J..

[30] Garnier P., Viateau V., Manassero M., Maurice E. (2022). Surgically treated congenital cleft palate in a 4-month-old kitten: Medium-term clinical and CT assessment. J. Feline Med. Surg. Open Rep..

[31] Henschele W.P. (1959). Cleft palate in lions of one litter. J. Amer. Vet. Med. Assoc..

[32] Scott J.H., Prophett A.S. (1955). Histologic investigation of cleft palate in tiger, dog and man. J. Dent. Res..

[33] McMichael L., McLean J., Taylor J., Martinez Y., Meers J. (2023). Cleft Palate Syndrome in the Endangered Spectacled Flying Fox (*Pteropus conspicillatus*): Implications for Conservation and Comparative Research. Vet. Sci..

[34] Halder A., Pahi J., Pradhan M., Pandey A., Gujral R., Agarwal S. (1998). Iniencephaly: A Report of 19 Cases. Indian Pediatr..

[35] Moore L. (2010). Anencephaly. J. Diagn. Med. Sonogr..

[36] Farré Mariné A., Pumarola M., Luján Feliu-Pascual A. (2022). Polysulfone tailor-made implant for the surgical correction of a frontoparietal meningoencephalocoele in a cat. J. Feline Med. Surg. Open Rep..

[37] Sponenberg D.P., Graf-Webster E. (1986). Hereditary meningoencephalocele in *Burmese cats*. J. Hered..

[38] Butterfield S., Garcia-Gonzalez B., Driver C.J., Rusbridge C. (2020). Limited dorsal myeloschisis in three cats: A distinctive form of neural tube defect. J. Feline Med. Surg. Open Rep..

[39] Lyons L.A., Erdman C.A., Grahn R.A., Hamilton M.J., Carter M.J., Helps C.R., Alhaddad H., Gandolfi B. (2016). Aristaless-Like Homeobox protein 1 (ALX1) variant associated with craniofacial structure and frontonasal dysplasia in *Burmese cats*. Dev. Biol..

[40] Muennich A. Congenital and hereditary diseases to be diagnosed in the kitten. Proceedings of the XIII Congreso de Especialidades Veterinarias.

[41] Padmanabhan R. (2006). Etiology, pathogenesis and prevention of neural tube defects. Congenit. Anom..

[42] Burren K.A., Savery D., Massa V., Kok R.M., Scott J.M., Blom H.J., Copp A.J., Greene N.D.E. (2008). Gene–environment interactions in the causation of neural tube defects: Folate deficiency increases susceptibility conferred by loss of Pax3 function. Hum. Mol. Genet..

[43] Yu Y., Wang F., Bao Y., Lu X., Quan L., Lu P. (2014). Association between MTHFR gene polymorphism and NTDs in Chinese Han population. Int. J. Clin. Exp. Med..

[44] Stover P.J., MacFarlane A.J., Field M.S. (2015). Bringing clarity to the role of MTHFR variants in neural tube defect prevention2. Am. J. Clin. Nutr..

[45] Momb J., Lewandowski J.P., Bryant J.D., Fitch R., Surman D.R., Vokes S.A., Appling D.R. (2013). Deletion of Mthfd1l causes embryonic lethality and neural tube and craniofacial defects in mice. Proc. Natl. Acad. Sci. USA.

[46] Di Pietro E., Sirois J., Tremblay M.L., MacKenzie R.E. (2002). Mitochondrial NAD-Dependent Methylenetetrahydrofolate Dehydrogenase-Methenyltetrahydrofolate Cyclohydrolase Is Essential for Embryonic Development. Mol. Cell. Biol..

[47] Narisawa A., Komatsuzaki S., Kikuchi A., Niihori T., Aoki Y., Fujiwara K., Tanemura M., Hata A., Suzuki Y., Relton C.L. (2012). Mutations in genes encoding the glycine cleavage system predispose to neural tube defects in mice and humans. Hum. Mol. Genet..

[48] Pangilinan F., Molloy A.M., Mills J.L., Troendle J.F., Parle-McDermott A., Signore C., O’Leary V.B., Chines P., Seay J.M., Geiler-Samerotte K. (2012). Evaluation of common genetic variants in 82 candidate genes as risk factors for neural tube defects. BMC Med. Genet..

[49] Piedrahita J.A., Oetama B., Bennett G.D., van Waes J., Kamen B.A., Richardson J., Lacey S.W., Anderson R.G.W., Finnell R.H. (1999). Mice lacking the folic acid-binding protein Folbp1 are defective in early embryonic development. Nat. Genet..

[50] Shaw G.M., Lu W., Zhu H., Yang W., Briggs F.B.S., Carmichael S.L., Barcellos L.F., Lammer E.J., Finnell R.H. (2009). 118 SNPs of folate-related genes and risks of spina bifida and conotruncal heart defects. BMC Med. Genet..

[51] Okano M., Bell D.W., Haber D.A., Li E. (1999). DNA Methyltransferases Dnmt3a and Dnmt3b Are Essential for De Novo Methylation and Mammalian Development. Cell.

[52] Fukuda T., Tokunaga A., Sakamoto R., Yoshida N. (2011). Fbxl10/Kdm2b deficiency accelerates neural progenitor cell death and leads to exencephaly. Mol. Cell. Neurosci..

[53] Vega R.B., Matsuda K., Oh J., Barbosa A.C., Yang X., Meadows E., McAnally J., Pomajzl C., Shelton J.M., Richardson J.A. (2004). Histone Deacetylase 4 Controls Chondrocyte Hypertrophy during Skeletogenesis. Cell.

[54] Bijok J., Dąbkowska S., Kucińska-Chahwan A., Massalska D., Nowakowska B., Gawlik-Zawiślak S., Panek G., Roszkowski T. (2023). Prenatal diagnosis of acrania/exencephaly/anencephaly sequence (AEAS): Additional structural and genetic anomalies. Arch. Gynecol. Obstet..

[55] Rull R.P., Ritz B., Shaw G.M. (2006). Neural Tube Defects and Maternal Residential Proximity to Agricultural Pesticide Applications. Am. J. Epidemiol..

[56] Baldo C., Campaña H., Gili J., Poletta F., Lopez Camelo J. (2008). Anencephaly and residence near textile industries: An epidemiological case-control study in South America. BAG J. Basic Appl. Genet..

[57] Tong M., Yu J., Liu M., Li Z., Wang L., Yin C., Ren A., Chen L., Jin L. (2021). Total mercury concentration in placental tissue, a good biomarker of prenatal mercury exposure, is associated with risk for neural tube defects in offspring. Environ. Int..

[58] DeSesso J., Jacobson C., Scialli A., Farr C., Holson J. (1998). An assessment of the developmental toxicity of inorganic arsenic 11Armand Lione, Ph.D., served as guest editor for this submission. Reprod. Toxicol..

[59] Willhite C.C. (1981). Arsenic-induced axial skeletal (dysraphic) disorders. Exp. Mol. Pathol..

[60] Stånge L., Carlström K., Eriksson M. (1978). Hypervitaminosis a in early human pregnancy and malformations of the central nervous system. Acta Obstet. Gynecol. Scand..

[61] Graham J.M., Ferm V.H. (1985). Heat- and Alcohol-Induced Neural Tube Defects: Interactions with Folate in a Golden Hamster Model. Pediatr. Res..

[62] Cagnotti G., Sammartano F., Bertone I., Capucchio M.T., Nicola I., Sacchi P., Bellino C., D’Angelo A. (2019). Imaging and genetic investigations of neural tube defect in a calf: Case report and review of the literature. J. Vet. Diagn. Investig..

[63] Copp A.J., Stanier P., Greene N.D.E. (2013). Neural tube defects: Recent advances, unsolved questions, and controversies. Lancet Neurol..

[64] Gonzales K.L., Famula T.R., Feng L.C., Power H.M.N., Bullis J.M. (2021). Folic acid supplementation does not decrease stillbirths and congenital malformations in a guide dog colony. J. Small Anim. Pract..

[65] Domosławska A., Jurczak A., Janowski T.E. (2013). Oral folic acid supplementation decreases palate and/or lip cleft occurrence in *Pug* and *Chihuahua* puppies and elevates folic acid blood levels in pregnant bitches. Pol. J. Vet. Sci..

[66] Zaganjor I., Sekkarie A., Tsang B.L., Williams J., Razzaghi H., Mulinare J., Sniezek J.E., Cannon M.J., Rosenthal J. (2016). Describing the Prevalence of Neural Tube Defects Worldwide: A Systematic Literature Review. PLoS ONE.

[67] Kıymaz N., Yılmaz N., Demir İ., Keskin S. (2010). Prognostic Factors in Patients with Occipital Encephalocele. Pediatr. Neurosurg..

[68] Zada G., Lopes M.B.S., Mukundan S., Laws E., Zada G., Lopes M.B.S., Mukundan S., Laws E.R. (2016). Meningoceles and Encephaloceles. Atlas of Sellar and Parasellar Lesions: Clinical, Radiologic, and Pathologic Correlations.

[69] Coulibaly O., Sogoba Y., Kanikomo D., Dama M., Camara M.A., Diallo O. (2016). Giant occipital meningohydroencephalocele in an adult: Another historical case in neural tube defects. Neurochirurgie.

[70] Kopke M.A., Jack M.W., Baltzer W.I., Wightman P.F., Gal A. (2019). Dermoid sinus type VI associated with spina bifida and tethered cord syndrome in a French Bulldog. J. Vet. Diagn. Investig..

[71] Kiviranta A.M., Lappalainen A.K., Hagner K., Jokinen T. (2011). Dermoid sinus and spina bifida in three dogs and a cat. J. Small Anim. Pract..

[72] Takahashi K., Kimura S., Chambers J.K., Nakano Y., Ishikawa T., Maeda S., Kamishina H. (2022). Case Report: Surgical Treatment of Type IV Spinal Dermoid Sinus in a Shiba Inu. Front. Vet. Sci..

[73] Wolf Z.T., Brand H.A., Shaffer J.R., Leslie E.J., Arzi B., Willet C.E., Cox T.C., McHenry T., Narayan N., Feingold E. (2015). Genome-Wide Association Studies in Dogs and Humans Identify ADAMTS20 as a Risk Variant for Cleft Lip and Palate. PLoS Genet..

[74] Juriloff D.M., Harris M.J. (2008). Mouse genetic models of cleft lip with or without cleft palate. Birth Defects Res. Part A Clin. Mol. Teratol..

[75] Gebuijs I.G.E., Raterman S.T., Metz J.R., Swanenberg L., Zethof J., Van den Bos R., Carels C.E.L., Wagener F.A.D.T.G., Von den Hoff J.W. (2019). Fgf8a mutation affects craniofacial development and skeletal gene expression in zebrafish larvae. Biol. Open.

[76] Moura E., Pimpão C.T. (2017). Cleft Lip and Palate in the Dog: Medical and Genetic Aspects.

[77] Lobodzinska A., Gruszczynska J., Max A., Jan Bartyzel B., Mikula M., Mikula I., Grzegrzolka B. (2014). Cleft palate in the domestic dog Canis Lupus Familiaris—Etiology, pathophtsiology, diagnosis, prevention and treatment. Acta Sci. Pol. Zootech..

[78] Mahajan T., Ganguly S., Saroj (2015). Embryological development of gastrointestinal tract in animals. Indian J. Sci. Res. Technol..

[79] Bhatia A., Shatanof R.A., Bordoni B. (2023). Embryology, Gastrointestinal.

[80] Lejeune B., Miclard J., Stoffel M.H., Meylan M. (2010). Intestinal Atresia and Ectopia in a Bovine Fetus. Vet. Pathol..

[81] Jaskwhich D., Ali R.M., Patel T.C., Green D.W. (2000). Congenital scoliosis. Curr. Opin. Pediatr..

[82] Eid T., Ghostine B., Kreichaty G., Daher P., Ghanem I. (2013). Congenital costo-vertebral fibrous band and congenital kyphoscoliosis: A previously unreported combination. Eur. Spine J..

[83] Ryan R., Gutierrez-Quintana R., ter Haar G., De Decker S. (2017). Prevalence of thoracic vertebral malformations in *French bulldogs*, *Pugs* and *English bulldogs* with and without associated neurological deficits. Vet. J..

[84] Havlicek M., Mathis K.R., Beck J.A., Allan G.S. (2009). Surgical management of vertebral malformation in a *Manx cat*. J. Feline Med. Surg..

[85] Inglez de Souza M.C.C.M., Ryan R., ter Haar G., Packer R.M.A., Volk H.A., De Decker S. (2018). Evaluation of the influence of kyphosis and scoliosis on intervertebral disc extrusion in *French bulldogs*. BMC Vet. Res..

